# Multidrug-Resistant *Acinetobacter baumannii*

**DOI:** 10.3201/eid1101.040001

**Published:** 2005-01

**Authors:** Aharon Abbo, Shiri Navon-Venezia, Orly Hammer-Muntz, Tami Krichali, Yardena Siegman-Igra, Yehuda Carmeli

**Affiliations:** * Tel Aviv Sourasky Medical Center, Tel Aviv, Israel

**Keywords:** Acinetobacter baumannii, multidrug resistance, nosocomial infections, hospital epidemiology, antibiotic resistance, molecular typing, risk factors, research

## Abstract

To understand the epidemiology of multidrug-resistant (MDR) *Acinetobacter baumannii* and define individual risk factors for MDR*,* we used epidemiologic methods, performed organism typing by pulsed-field gel electrophoresis (PFGE), and conducted a matched case-control retrospective study. We investigated 118 patients, on 27 wards, in whom MDR *A*. *baumannii* was isolated from clinical cultures. Each case-patient had a control without MDR *A*. *baumannii* and was matched for hospital length of stay, ward, and calendar time. The epidemiologic investigation found small clusters of up to 6 patients each with no common identified source. Ten different PFGE clones were found, of which 2 dominated. The PFGE pattern differed within temporospatial clusters, and antimicrobial drug susceptibility patterns varied within and between clones. Multivariate analysis identified the following significant risk factors: male sex, cardiovascular disease, having undergone mechanical ventilation, and having been treated with antimicrobial drugs (particularly metronidazole). Penicillins were protective. The complex epidemiology may explain why the emergence of MDR *A. baumannii* is difficult to control*.*

## Abstract 2

A case-control, epidemiologic, and molecular study of nosocomial MDR *A. baumannii* showed the existence of multiple clones and a complex epidemiologic pattern.

*Acinetobacter baumannii* has emerged as an important nosocomial pathogen (*1*–*5*). Hospital outbreaks have been described from various geographic areas (*6*–*9*), and this organism has become endemic in some of them. The role of the environmental contamination in the transmission of nosocomial infections in general and in *A. baumannii* infections in particular is well recognized (*10*,*11*). *A. baumannii* does not have fastidious growth requirements and is able to grow at various temperatures and pH conditions (*12*). The versatile organism exploits a variety of both carbon and energy sources. These properties explain the ability of *Acinetobacter* species to persist in either moist or dry conditions in the hospital environment, thereby contributing to transmission (*13*,*14*). This hardiness, combined with its intrinsic resistance to many antimicrobial agents, contributes to the organism’s fitness, enables it to spread in the hospital setting.

The nosocomial epidemiology of this organism is complex. Villegas and Hartstein reviewed *Acinetobacter* outbreaks occurring from 1977 to 2000 and hypothesized that endemicity, increasing rate, and increasing or new resistance to antimicrobial drugs in a collection of isolates suggest transmission. These authors suggested that transmission should be confirmed by using a discriminatory genotyping test (*15*). The importance of genotyping tests is illustrated by outbreaks that were shown by classic epidemiologic methods and were thought to be caused by a single isolate transmitted between patients; however, when molecular typing of the organisms was performed, a more complex situation of multiple unrelated strains causing the increasing rates of infections by *A. baumannii* was discovered (*16*–*18*).

Almost 25 years ago, researchers observed acquired resistance of *A. baumannii* to antimicrobial drugs commonly used at that time, among them aminopenicillins, ureidopenicillins, first‑ and second-generation cephalosporins, cephamycins, most aminoglycosides, chloramphenicol, and tetracyclines (*19*). Since then, strains of *A. baumannii* have also gained resistance to newly developed antimicrobial drugs. Although multidrug-resistant (MDR) *A. baumannii* is rarely found in community isolates, it became prevalent in many hospitals (*20*). MDR *A. baumannii* has recently been established as a leading nosocomial pathogen in several Israeli hospitals, including our institution (*21*,*22*). Several locally contained small outbreaks of MDR *A.* *baumannii* occurred in our institution during the late 1990s. In 1999, however, the incidence of MDR *A. baumannii* isolation had doubled compared to the previous 2 years, and the organism became endemic in many wards (unpub. data).

The likelihood of isolation of *A.* *baumannii* from a hospitalized patient is related to temporospatial (extrinsic, ecologic characteristics) factors such as colonization pressure (*23*), nurse-to-patient ratio, and other ward characteristics and to individual patient risk factors (characteristics). The current study was designed to examine the occurrence and spread of *A.* *baumannii* within our institution, as well as to define individual risk factors for isolation of this organism.

## Methods

### Hospital Setting, Data Collection, and Microbiologic Testing

This study was performed at the Tel-Aviv Sourasky Medical Center, Israel, a 1,200–bed tertiary care teaching hospital with 70,000 admissions annually. Approximately 82,500 clinical microbiologic cultures are processed annually. We designed this as a matched case-control study to identify the individual risk factors for having MDR *A. baumannii.* We also performed epidemiologic investigations and genetic typing of the organisms to clarify the spread of this nosocomial pathogen.

Case-patients were defined as patients from whom MDR *A. baumannii* was isolated from any clinical culture (not surveillance cultures) during a 6–month period, from January 1, 2001, to June 30, 2001. A control patient was matched to each study patient on temporospatial factors as previously described (*24*). Briefly, controls were randomly chosen from the list of patients who stayed on the same ward in the same calendar month as the matched case-patient and who were hospitalized for at least the same number of days by the day the culture yielded MDR *A. baumannii* in the study patient. Controls were not MDR *A. baumannii* positive (i.e., the patient’s samples were cultured, and either non-MDR *A. baumannii* or no *A. baumannii* was isolated, or the patient’s samples were never cultured). Random control selection was performed by creating a list of all possible controls, assigning each candidate a random number, and choosing the highest random number (without replacement).

Case-patients and control patients were included only once in the study. Data were collected from the patients’ records and from hospital computerized databases into a pre-prepared electronic questionnaire (Microsoft Access, Microsoft Corp., Redmond, WA, USA). The parameters registered for each patient (case-patients and controls) were age, sex, habits of smoking and alcohol consumption before hospitalization, cause and ward of hospitalization, transfer from another institution or ward within our institution, intensive care unit (ICU) stay, underlying disorders, immunosuppressive therapy, severity of illness as defined by the McCabe score (*25*), functional capacity and neurologic condition at time of isolation of A. *baumannii*, Foley catheter, invasive devices, surgery, mechanical ventilation, dialysis, infection, and antimicrobial drug therapy. Only variables occurring before inclusion in the study (culture day for case-patients and match day for controls) were analyzed as possible risk factors. *A.* *baumannii* was isolated from clinical specimens submitted to the microbiology laboratory and identified by using the Gram-Negative Identification Panel (Microscan, Dade Behring Inc., Sacramento, CA, USA). This system may not distinguish between closely related genotypic strains of *Acinetobacter*, and thus, some of these organisms may belong to these closely related strains. Susceptibilities were determined by automated microdilution broth testing (Neg/Urine Combo panel, Dade Behring Inc.). Resistance to imipenem and meropenem was confirmed by using Kirby-Bauer disk diffusion, according to the National Council for Clinical Laboratory Standards (NCCLS) guidelines. *A. baumannii* isolates were collected prospectively and stored at –70°C for further work-up.

### Analysis of Chromosomal DNA by Pulsed-Field Gel Electrophoresis (PFGE)

Isolates of our patients, when available, were kept frozen at –70ºC and genetically characterized with PFGE. DNA preparation and cleavage with 20 U of *Apa*I endonuclease (New England Biolabs, Beverly, MA, USA) were preformed as previously described (*26*). Electrophoresis was performed in a 1% agarose gel (BMA products) prepared and run in 0.5 x Tris-borate-EDTA buffer on a CHEF-DR III apparatus (Bio-Rad Laboratories, Hercules, CA, USA). The initial switch time was 5 s, the final switch time was 35 s, and the run time was 23 h at 6 V/cm. Gels were stained in ethidium bromide, destained in distilled water, and photographed by using a Bio-Rad GelDoc 2000 camera. DNA patterns were analyzed visually and by using Diversity software (Bio-Rad). PFGE DNA patterns were compared and interpreted according to the criteria of Tenover et al. (*27*). The obtained PFGE DNA patterns were used to cluster the clones of the *A. baumannii* clinical isolates that were included in the study*.*

## Definitions

We defined *A*. *baumannii* as MDR when the organism was resistant to all studied agents (including piperacillin/tazobactam, cefepime, ceftazidime, aztreonam, ciprofloxacin, gentamicin, tobramycin), but we allowed susceptibility to amikacin, ampicillin-sulbactam, imipenem, meropenem, and minocycline. Infection was defined according to the Centers for Disease Control and Prevention guidelines and modified to include community-acquired infections and to exclude asymptomatic bacteriuria (*28*).

Standard criteria were used to define underlying disorders. Disease was considered to be active if signs of disease were clinically apparent or if the patient received treatment for the disease. A patient was considered to be receiving immunosuppressive therapy if he had undergone chemotherapy within 3 weeks, if he had been treated with >20 mg of prednisone daily for >2 weeks before entering the study, or if he had recently received antirejection drugs or other immunosuppressive therapy.

Severity of illness due to comorbidities was defined according to the McCabe score (*25*). Functional capacity during the index hospitalization was divided into 3 categories: independent, needing help for activities of daily living, and bedridden. Renal failure was defined as a creatinine level >2 mg/dL. Neurologic function was categorized according to 3 conditions: full consciousness, confusional state or dementia, and unconscious.

For each patient included in the study, we noted whether a susceptible *A. baumannii* was isolated in any culture before isolation of the MDR strain. We noted the number of antimicrobial drugs that the patient received between the time of admission until inclusion in the study, and we recorded home antimicrobial drug therapy separately. Recent hospitalization was defined as hospital stay within 3 months of the index hospitalization. We noted any surgical procedure, mechanical ventilation, and invasive procedure that took place 1 month before the patient’s inclusion in the study.

### Statistical Analysis

Statistics were run in Stata version 7 (Stata Corp., College Station, TX). All analyses were matched to correspond to the study design. All variables were examined by univariate analysis with the McNemar test and paired Student *t* test. Variables with a p value <0.2 in the univariate analysis were included in the multivariate model. Risk factors were examined by using conditional logistic regression. A final model was built that included all the variables with a p value <0.2. Variables that were not retained in the model by this procedure were then tested for confounding by adding them 1 at a time to the model and examining their effects on the β coefficients. Variables which caused substantial confounding (change in β coefficient of >10%) were included in the final model. After constructing the explanatory model, the effect of exposure to antimicrobial agents (i.e., antimicrobial treatment before inclusion in the study) was examined by adding them to the model.

In addition to examining statistical significance and confounding, the effect modification between variables was evaluated by testing appropriate interaction terms for statistical significance. Colinearity was examined by replacing variables with each other and examining the effect on the model. All statistical tests were 2-tailed. A p value <0.05 was considered significant.

## Results

From January 1, 2001, to June 30, 2001, we identified 133 patients with a clinical culture of MDR *A.* *baumannii*. Four patients were not hospitalized in our institution (i.e., they were hospitalized elsewhere) and were excluded from the study. Charts were available for 120 case-patients, but no controls could be matched for 2 of them. Thus, 236 patients were included in the study (118 case-patients and their matched controls). Sites from which *A. baumannii* was initially isolated included respiratory tract 38 (32%), wounds 23 (19.5%), urine 22 (19%), blood 19 (16%), and sterile fluids and catheter tips 16 (13.5%).

### Epidemiology and PFGE Typing

**Figure 1:**
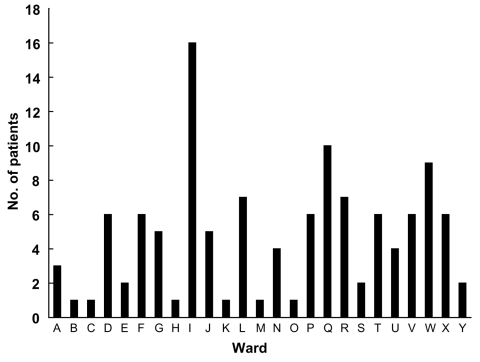
Distribution of case-patients according to ward.

Among the 118 case-patients, the first MDR *A. baumannii* in 104 (88%) was isolated after more than 72 h of hospitalization (mean 17.5 ± 23.7 days). Among the other 14 case-patients, 12 were admitted from another institution or had been hospitalized recently. No nosocomial origin was documented in 2 cases. *A. baumannii* was initially isolated in 27 different wards. Figure 1 shows the case distribution among them. A higher concentration of patients was clearly evident in 3 wards: the general ICU (ward “I”, 16 cases), and two internal medicine wards (ward Q, 10 cases, and ward W, 9 cases).

**Figure 2:**
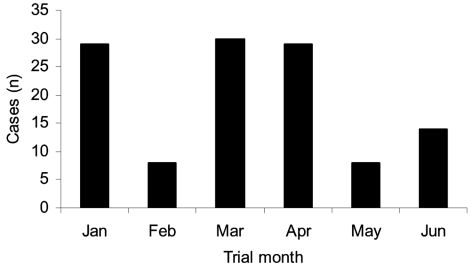
Monthly case distribution

The time distribution of new cases is presented in Figure 2. We did not find any aggregate of cases within a specific ward at any specific time. The occurrence during the months February, May, and June was lower than during January, March, and April. This circumstance is not explained by differences in infection control measures recommended; during the entire study period this included contact isolation of every patient from whom MDR *A. baumannii* was isolated and cohorting of case-patients if single patient rooms were not available. A statement was added to the culture result: “MDR organism; contact isolation is required.” Epidemiologic nurses checked with the ward to confirm that the patient was isolated. On certain floors surveillance (nose, forearms, armpits, and perirectal swabs) and environmental cultures (using swabs, contact plates, and direct culturing of fluids) were performed to try and identify a reservoir of organism. In this study, case-patients and controls were matched by ward and calendar time to focus on individual risk factors and not on differences between wards and temporal changes.

**Figure 3:**
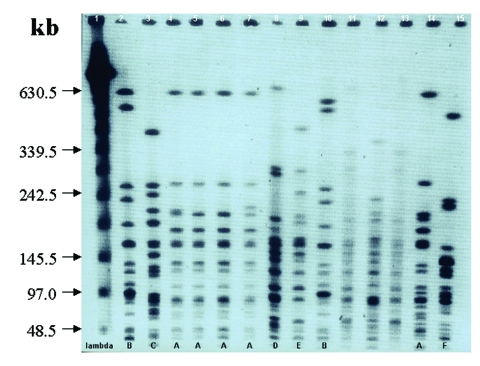
A typical pulsed-field gel electrophoresis analysis of selected isolates of A. *baumannii* restricted with *Apa*I. Lane 1 shows λ ladder used as molecular size marker. Lanes 11–13 are of strains not included in the trial. The gel shows 6 different clones of A. *baumannii*: 5 isolates belong to clone A and that 2 belong to clone B (the two dominant clones). Single isolates belonging to clones C, D, E, and F can be seen.

A total of 51 unique patient MDR strains were available for further study, and they were analyzed by using PFGE. We identified 10 distinct clones of *A. baumannii.* Figure 3 shows 6 different PFGE-defined clones, each having from 1 to 4 subtypes showing a 1- to 2-band difference. Two of the 10 different clones dominated: 22 case-patients had clone A and 10 case-patients had clone B, although no specific clone dominated in a specific ward but rather each clone was spread among several wards during the entire study period (Table 1). We also found various antimicrobial drug susceptibility phenotypes (all belonging to our definition of MDR) within each PFGE clone, but almost all cases of carbapenem resistance belonged to clone A.

**Table 1:** Characteristics of 51 strains available for PFGE typing, 2001*

Clone	No. of isolates	No. of wards	Months	Carbapenem resistance	Sulbactam resistance
A	22	14	January–June	16	1
B	10	8	February–July	1	
C	4	4	January–June	1	2
D	2	2	January, June		
E	2	1	January		
F	3	3	March, April		
I	5	4	February–April		2
Lone†	1	1	January		
Lone†	1	1	January		
Lone†	1	1	April		

*PFGE, pulsed-field gel electrophoresis. †Lone, unique clone; each had a different ward of isolation.

### Individual Risk Factors

The study patients’ characteristics are displayed in Table 2. Case-patients were similar to their matched control patients with respect to mean age (67.7 vs 64.4 years) and sex distribution (men, n = 71 [60%] vs controls, n = 59 [50%]). The groups were also similar in habits of smoking and alcohol consumption and in the occurrence of coexisting conditions of lung disease, diabetes, kidney, liver disease, malignancy, and posttransplantation condition. The groups differed in the prevalence of ischemic heart disease: study case-patients 69% vs. controls 52% (OR 2.33, p = 0.006).

**Table 2:** Group comparison: patients’ characterization and possible risk factors for *Acinetobacter* *baumannii* isolation*†

	Cases, n (%)	Controls, n (%)	OR	95% CI	p value
Demographic parameters					
Average age, y (SD)	67.7 (16.42)	64.4 (19.15)	1.012	0.996–1.03	0.134
Female	47 (40)	59 (50)			
Male	71 (60)	59 (50)	1.48	0.89–2.45	0.13
Smoking	35 (30)	43 (36)	0.72	0.41–1.27	0.26
Alcohol usage	7 (6)	5 (4)	1.5	0.42–5.31	0.53
Admission from home	23 (20)	12 (10)	2.12	0.96–4.76	0.065
Concomitant diseases					
Ischemic heart disease	82 (69)	61 (52)	2.33	1.27–4.27	0.006
Lung disease	59 (50)	50 (42)	1.578	0.82–2.72	0.183
Diabetes	39 (33)	28 (24)	1.578	0.88-2.8	0.119
Liver disease	10 (8)	18 (15)	0.555	0.25–1.2	0.136
Kidney disease	35 (30)	27 (23)	1.388	0.76–2.54	0.288
Posttransplantation	7 (6)	3 (3)	2.333	0.6–9.02	0.22
Malignancy	35 (30)	38 (32)	0.923	0.53–1.6	0.777
Clinical parameters					
Unconsciousness	40 (43)	26 (22)	0.706	0.48–1.02	0.069
Bedridden	88 (75)	79 (76)	1.45	0.82–2.56	0.201
In-house dialysis	11 (9)	4 (3)	2.5	0.78–7.97	0.121
Mechanical ventilation	70 (59)	47 (40)	2.916	1.51–5.61	0.001
Admission in last 3 months	56 (47)	52 (44)	1.148	0.68–1.92	0.6
ICU stay	34 (92)	35 (30)	0.984	0.94–1.02	0.473
Immunosuppression treatment	29 (25)	27 (32)	1.095	0.9–1.97	0.763
Major surgery	33 (28)	33 (28)	1	0.51–1.95	1
Isolation of susceptible *Acinetobacter* before inclusion	6 (5)	6 (5)	1		1
Antimicrobial treatment					
Home antimicrobial treatment	16 (41)	6 (5)	2.666	1.04–6.81	0.04
In-house antimicrobial treatment	104 (88)	96 (81)	1.727	0.82–3.63	0.149
Average number of antimicrobial agents (SD)	3.025 (2)	2.97 (2)	1.015	1	0.847
Penicillin administration‡	42 (63)	54 (46)	0.647	0.37–1.1	0.112
Cephalosporin use (1st, 2nd generation)	31 (26)	34 (92)	0.9	0.476–1.701	0.746
3rd generation cephalosporin use	53 (54)	42 (63)	1.631	0.92–2.88	0.093
4th-generation cephalosporin use	17 (41)	17 (14)	1	0.49–2.04	1
Macrolides	21 (18)	11 (9)	2.25	0.97–5.17	0.056
Metronidazole	48 (14)	35 (30)	1.933	1.03–3.6	0.038
Gentamicin	23 (19)	27 (32)	0.777	0.38–1.56	0.481
Amikacin	12 (10)	19 (16)	0.5	0.2–1.24	0.134
Clindamycin	7 (6)	8 (7)	0.875	0.32–2.41	0.796
Vancomycin	22 (19)	22 (19)	0.944	0.48–1.83	0.866
Carbapenem	10 (8.47)	10 (8.47)	0.875	0.32–2.41	0.796
Invasive procedures					
Central line	69 (58)	70 (59)	0.958	0.54–1.7	0.884
Arterial line	35 (30)	34 (29)	0.933	0.45–1.93	0.85
Foley catheter	96 (81)	76 (64)	2.42	1.3–4.52	0.005
Other bedside procedures§	63 (53)	78 (67)	0.531	0.29–0.95	0.035

*OR, odds ratio; CI, confidence interval; SD, standard deviation; ICU, intensive care unit. †All variables were recorded up to the time of inclusion in the study. ‡Including semisynthetic penicillin and β-lactamase‑containing products. §Including tracheostomy, bedside débridement, chest tube insertion, and gastrointestinal endoscopy.

Hospital events (before study entry) differed between case-patients and controls. Case-patients were more likely to have received mechanical ventilation (OR 2.9, p = 0.001), to be treated with metronidazole (OR 1.9, p = 0.038), and to have a Foley catheter (OR 2.42, p = 0.005). They were less likely to have had another bedside surgical procedure before the isolation of *A. baumannii* (OR 0.53, p = 0.035).

Several variables tended to be more associated with case-patients, but the values did not reach statistical significance: admission from another institute (OR 2.1, p = 0.06), unconsciousness (OR 0.706, p = 0.07), and previous use of third-generation cephalosporin (OR 1.63, p = 0.093) and of macrolides (OR 2.25, p = 0.056). A matched multivariate model, adjusted for the hospital length of stay, was developed by using conditional logistic regression (Table 3). The variables that were identified by this model as being significant risk factors for MDR *A. baumannii* were male sex (OR 3.8, p = 0.002), ischemic heart disease (OR 3.3, p = 0.005), mechanical ventilation (OR 6.2, p < 0.001), and home antimicrobial drug use (OR 4.7, p = 0.018). Two agents used in the hospital were associated with MDR *A. baumannii*: metronidazole was identified as a risk factor (OR 2.3, p = 0.018), and the penicillin group was identified as having a protective effect (OR = 0.38, p = 0.029).

**Table 3:** Multivariate analysis for risk factors for *Acinetobacter* baumannii*†

Parameter‡	OR	95% CI	p value
Male sex	3.84	1.63–8.99	0.002
Ischemic heart disease	3.35	1.44–7.77	0.005
Mechanical ventilation	6.27	2.27–17.33	<0.001
Penicillin use§	0.38	0.16–0.90	0.029
Metronidazole use	2.33	0.98–5.83	0.071
Any home antimicrobial drug treatment	4.74	1.31–17.15	0.018

*OR, odds ratio; CI, confidence interval. †Adjusted for length of hospital stay prior to entry to the study. ‡All parameters had been present before *A.* *baumannii* identification. §Including semisynthetics with or without a β-lactamase inhibitor (never sulbactam).

## Discussion

We sought to understand the epidemiology of MDR *A. baumannii* and to define the individual risk factors for acquiring this infectious agent. Our findings illustrated its complex epidemiology and delineated individual risk factors. The complex epidemiology may explain the difficulties encountered in controlling the emergence of this nosocomial pathogen.

Almost all cases of MDR *A. baumannii* in our study were hospital acquired: 88% were acquired in our institution during the index hospitalization, and 10% were imported into the hospital by patients with recent exposure to the healthcare system. The MDR *A. baumannii* strains isolated in our institution belonged to multiple PFGE clones: 50% of the isolates that were typed belonged to 2 dominant clones, and the other, nondominant clones caused few cases each.

Clones did not cluster in place (i.e., hospital location) or in time. Moreover, when an increase in incidence was observed in a certain ward, the increase was not associated with a single clone, and up to 4 different clones were present concomitantly in a ward. We also found antimicrobial susceptibility profile variation within clones and similarities between clones, which showed that susceptibility pattern was not a useful marker for clonality. Carbapenems resistance occurred in 75% of the isolates belonging to 1 of the 2 dominant clones (clone A) but was rare among other clones. This finding illustrates well the complexity of the epidemiology of this nosocomial pathogen. Even with molecular typing data, determining the modes of spread of this organism was difficult, partly because we did not have a complete collection of the isolates. Despite our expending extensive effort, we were unable to determine the source of these resistant strains. Although we believe that patient-to-patient transmission through contaminated hands of healthcare workers and fomites is the main route by which these MDR organisms spread, the combined epidemiologic and molecular data did not directly support this hypothesis. The lack of evidence for patient-to-patient spread in our study may be related to transferring patients between wards and the presence of a substantial number of undetected carriers (the “submerged iceberg phenomenon”) who spread the bacteria. Alternative explanations, such as repeated entry (import) of the same clone to the hospital ecosystem at various times and locations (e.g., from an disease-endemic institution or contaminated supply or food) must be considered as well.

The individual risk factors for isolation of MDR *A.* *baumannii* that were identified by the multivariate analysis were male sex, underlying comorbidity of ischemic heart disease, mechanical ventilation, and antimicrobial drug treatment. The finding of male sex and of ischemic heart disease being risk factors for carriage of and infection with resistant gram-negative bacilli had also been observed by our group, as well as by others for carriage of extended spectrum β-lactamase (ESBL)‑producing *Enterobacteriaceae* and *Pseudomonas aeruginosa* (*29*–*31*). We hypothesize that these associations may be related to the following factors: 1) patient-to-patient transmission within multipatient rooms (patients who are segregated by sex and need for intensive monitoring); 2) use of certain nonantimicrobial medication, such as calcium channel blockers, which may predispose for adherence or invasiveness by affecting the host or the pathogens (*32*,*33*); and 3) hormonal or other sex differences which may predispose a person for colonization and infection. These hypotheses are currently being studied in our facilities. The multivariate analysis did not identify admission from another institution as a significant risk factor. This probably relates to the small number (and proportion) of patients admitted from other institutions who were identified to be carriers of MDR *A.* *baumannii*. These few patients may, however, have played an important role to the entrance of new clones and the spread of the organisms within our institution. Moreover, case-patients that are not detected may still be important in the spread of these organisms. Overall, we believe that the identified risk factors represent both severity of the patient’s condition, use of invasive devices, and effect on the normal flora, all of which promote MDR *A.* *baumannii* colonization, growth, and invasiveness.

The administration of penicillin had a protective effect against isolation of MDR *A. baumannii*. This protective effect was significant after confounding by multivariate analysis was controlled for. Penicillins lack activity against these MDR strains, and the protective effect cannot relate to sulbactam, since a sulbactam combination is seldom used in our institution. To the best of our knowledge, such an effect has not been observed previously. Specific penicillins may possibly cause specific changes in the microflora that oppose colonization and growth of *Acinetobacter* spp., but the validity of this observation awaits further research. As for many other resistant organisms, metronidazole was a significant risk factor for MDR *A.* *baumannii*, likely because of its effects on the competitive normal intestinal flora. The observation that carbapenem resistance was much more frequent in the dominant clone could suggest that this phenotype may have contributed to the evolutionary success of the clone.

A previous study clearly demonstrated that the epidemiology and risk factors may vary for different clones (*17*). This finding may lead to a dilution of effects and even to opposing effects by some risk factors. In our study, we did not analyze clone-specific risk factors because we did not believe that we truly had an epidemic clone and because the number of patients affected by each clone was too small to allow a statistically significant comparison.

Temporospatial factors, although they undoubtedly have an important role in the spread of resistant organisms, were not within the scope of this study. We controlled for these factors by the study design, i.e., matching by hospital location, length of stay before inclusion in the study, and calendar time. Confounding may, however, have been introduced to our study by factors for which we did not control, such as residing in a multipatient room next to a patient with MDR *A. baumannii*.

We used risk set sampling (by matching for time at risk) but did not allow case-patients to be eligible to be controls before to becoming cases. Since no clustering in time and place occurred, and controls were chosen from 35,000 admitted patients, this method of sampling should not have yielded biased results.

Despite the large number of cases that we identified, we were unable to understand the mode of spread and the reason for emergence of these organisms in our institution. This fact may be because only some of the isolates were available for typing or because of the complex mode of spread in our hospital. Further study will be required to more fully understand the intricate phenomenon of MDR *A. baumannii* spread.
